# Detusking Fence-Breaker Elephants as an Approach in Human-Elephant Conflict Mitigation

**DOI:** 10.1371/journal.pone.0091749

**Published:** 2014-03-10

**Authors:** Matthew Mutinda, Geoffrey Chenge, Francis Gakuya, Moses Otiende, Patrick Omondi, Samuel Kasiki, Ramón C. Soriguer, Samer Alasaad

**Affiliations:** 1 Kenya Wildlife Service, Nairobi, Kenya; 2 Lewa Wildlife Conservancy, Private Bag Isiolo, Kenya; 3 Estación Biológica de Doñana, Consejo Superior de Investigaciones Científicas (CSIC), Sevilla, Spain; 4 Institute of Evolutionary Biology and Environmental Studies (IEU), University of Zürich, Zürich, Switzerland; Institut Pluridisciplinaire Hubert Curien, France

## Abstract

**Background:**

Human-elephant conflict (HEC) is a recurring problem that appears wherever the range of elephants and humans overlap. Different methods including the use of electric fences are used worldwide to mitigate this conflict. Nonetheless, elephants learn quickly that their tusks do not conduct electricity and use them to break down fences (fence-breakers).

**Methodology/Principal Findings:**

In Lewa Wildlife Conservancy, Kenya, destructive elephants (*Loxodonta africana*) were monitored between 2010 and 2013. The fence-breaking rate reached four incidents (fence-breaking) per elephant per 100 days. Ten bull males and 57 females were identified as fence-breakers. The bulls were involved in 85.07% and the females in 14.93% of incidents. The Kenya Wildlife Service approved detusking (partial cutting of tusks) in four of the 10 fence-breakers as a way of preventing them from breaking down fences, thereby mitigating HEC in the Conservancy. The result of the detusking was a drastic six-fold reduction in damage to fences (range: 1.67 to 14.5 times less fence-breaking) by the four worst fence-breaker elephants, because with trimmed tusks elephants lack the tools to break down fences. Detusking could not totally eliminate fence destruction because, despite lacking their tools, elephants can still destroy fences using their heads, bodies and trunks, albeit less effectively. On the other hand, apart from inherent aesthetic considerations, the detusking of elephants may have certain negative effects on factors such as elephants' social hierarchies, breeding, mate selection and their access to essential minerals and food.

**Conclusions:**

Elephant detusking seems to be effective in drastically reducing fence-breaking incidents, nonetheless its negative effects on behaviour, access to food and its aesthetical consequences still need to be further studied and investigated.

## Introduction

As the human population expands, the natural territories of wild animals are displaced and animal and human populations coincide with increasing frequency. Although physical interaction between wild animals and humans may benefit some wild populations [1], [2], this overlap is in general regarded as potentially destructive for both humans and wild animals. Reducing conflicts between wildlife and people is today regarded as a top priority in conservation, particularly in landscapes where high densities of people and wildlife co-occur [3].

Kenya has one of the fastest growing human populations in the world and the number of new inhabitants increases by approximately one million per year. This rise in the country's human population had led to increased land pressure in areas previously used exclusively by wildlife. Today, infrastructure projects are a common sight in areas that were once areas of wildlife dispersal. Similarly, nomadic pastoral communities have settled near protected areas, above all to guarantee pasture for their herds but in some cases to cultivate the land. This has led to increased and continuous human-wildlife conflicts (HWC) [4]–[6].

HWC has a wide range of consequences that include the loss of human life, human threats/obstruction, crop destruction, damage to property, habitat destruction, injuries to people as well as wildlife, and livestock predation by wildlife [7]–[11]. Reports from Kenya indicate that out of the 9,299 HWC cases reported over the last 10 years, 5,052 (54%) consist of incidents of human-elephant conflict (HEC) (KWS unpublished data).

HEC consists of any human-elephant interaction that has negative effects on human social, economic or cultural life, on elephant conservation or on the environment [2], [12]. It is a chronic problem that occurs throughout the world wherever elephants and people share the same habitat. HEC is often regarded as the major threat to the long-term survival of the African elephant [2], even if the most important drivers of fluctuations in elephant populations are in fact poaching and habitat destruction [13].

To mitigate this conflict several traditional (noise makers, catapults, rocks, burning sticks, barrier construction), conventional (shooting to scare, electric fencing, translocation of problem elephants) and locally adapted deterrence methods, along with a number of changes in land-use planning and policy at national scale, have been put into practice to reduce local levels of HEC [11], [14]–[22].

Electric fences are costly to build and maintain but are recognised as a potential means of reducing conflicts since they prevent access to vulnerable land and enable people and elephants to be separated at landscape scale [23], [24]. The effectiveness of electric fences in controlling elephant movements depends on a number of factors including design, number of strands, number of electrified wires, configuration and the effectiveness of both maintenance and responses to reports of fence-breaking animals [25].

By physically separating wildlife and humans, electric fencing can be an effective method of managing wildlife. Nonetheless, elephants learn quickly that their tusks do not conduct electricity and so use them to break down fences (fence-breakers) [25]. The Kenya Wildlife Service (KWS) approved the detusking (trimming of a part of their tusks) of some of the fence-breaker elephants in the Lewa Wildlife Conservancy (LWC) as a way of preventing these animals from breaking down fences and helping to mitigate HEC in the Conservancy. A tusk is a tooth with a very large nerve running part way down its centre and so trimming tusks makes the end of tusks far more sensitive to the electric current running through fence wires. Elephant tusks should be regarded as a vital part of this charismatic species – their size is a great indicator of an animal's genetic strength and viability and they play a vital tool in the duels that determine the dominant bull in a group. They are used to dig roots from the ground, strip the bark off trees for food and dig out essential minerals from the soil [26]. Furthermore, the aesthetic value of the tusks in relation to the tourist industry should not be underestimated [27].

The aim of this study was to provide the scientific and management communities with information regarding the demographic structure of fence-breaker elephants and the effectiveness of the detusking of four of the worst fence-breakers in LWC by the Kenya Wildlife Service as a means of mitigating HEC and the relative merits of this practice.

## Methods

### Lewa Wildlife Conservancy

Lewa Wildlife Conservancy (62,000 acres) lies between latitude 0°13′20″ N and longitude 37°27′51″ E in northern Kenya on the Laikipia plateau. At its northern-most point, it borders the foothills of Mount Kenya and has an altitudinal gradient ranging from 1,450 m.a.s.l. in the north to 2,300 m.a.s.l. in the south. Two permanent rivers cross Lewa and, together with an extensive swamp, form the lifeline of the people and wildlife in the Conservancy and in the more arid lowland areas in northern Kenya. The external boundary fence is 142-km long, 7-feet high, and has 12 strands of alternating live and earth wires. Certain zones in particular are also protected with two strands of live wires to preserve the woody vegetation needed by the endangered black rhino. The voltage of the two fences is maintained at 5.0–9.0 kV. The northern boundary fence has a gap through which animals migrate in and out of the Conservancy. The first fence was put up in 1984 at Ngare Sergoi rhino sanctuary in Lewa, while the main external fence was erected in 1990. There are about 500 Elephants (*Loxodonta africana*) in the Conservancy, about 150 (30%) males and 350 (70%) females. Age classes within each gender are 16% juveniles (≤5 years), 36% sub-adults (>5–10 years), and 48% adults (>10 years).

### Destructive elephants monitoring

**Figure 1:**
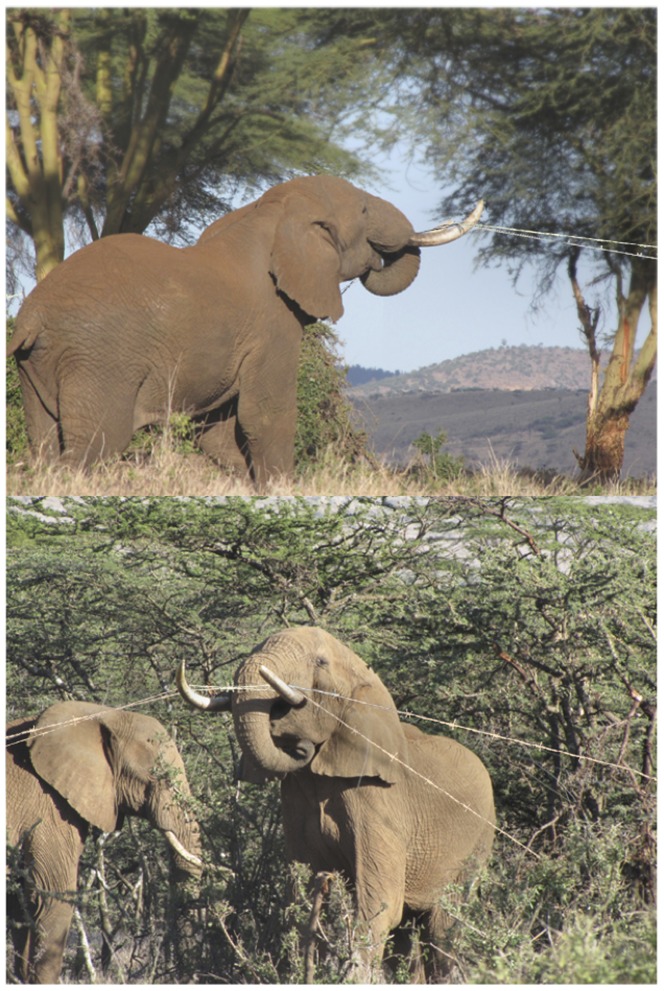
(Top) Fence-breaking elephant in Lewa Wildlife Conservancy attempting to snap two strands of wires by pushing them upwards. (Bottom) The partner elephant is busy crawling below the live wire. The photos were manipulated to highlight the wires, which were not very clear in the originals.

Destructive elephants were monitored between September 2010 and September 2013 to (i) identify individual elephants that damage fences and crops and to (ii) implement appropriate management strategies aimed at minimizing fence damage, which included the detusking of certain destructive elephants and the evaluation of the effects of this intervention. Damage to fences caused by elephants was reported by field security and fencing teams to the Lewa Radio Room and the Elephant Monitor. The Elephant Monitor, who is equipped with a motorbike, binoculars and a digital camera, visits all damaged points and gathers appropriate information, which includes photos of the elephants that damage the exclusion zones and the main boundary fences. These photos are subsequently compared with those in the database (Fig. 1) and new individuals not included in the database are added accordingly. The Monitor also notes the presence of other elephants that are not fence-breakers, but which presumably can access the area once the fence is broken down.

### Detusking of fence-breakers

**Figure 2:**
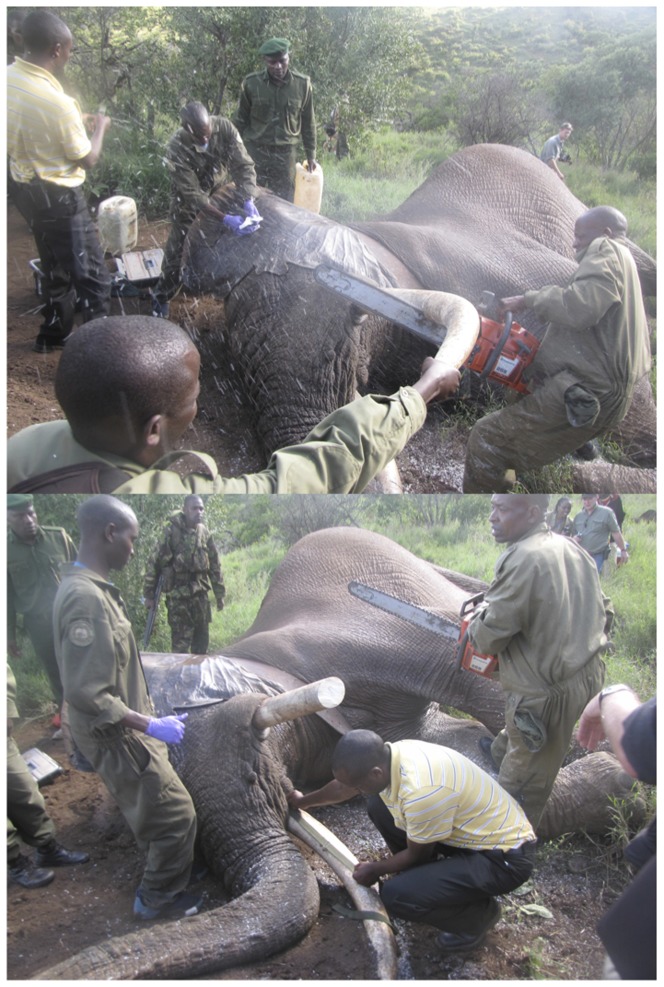
(Top) Elephant detusking using a power saw. (Bottom) Measuring the tusk to ensure that the nerve is not exposed after detusking (Lewa Wildlife Conservancy). The KWS vets and rangers in the photograph have given their written consent, as outlined in the PLOS consent form, for this photograph to be published.

Four of the most destructive elephants (the 10 males that caused the highest number of fence-breaking incidents out of the total 67 fence-breakers) were detusked: Flyn was detusked on 8 September 2011, Mashauri on 25 May 2012, Javet on 26 September 2012, and Right Notch on 27 November 2012. Elephants were darted on foot or from a vehicle using the Daniject darting system and 18 mg of etorphine hydrochloride (Norvatis, South Africa) mixed with 5000 iu (International Unit) of hyaluronidase (Kyron Laboratories, Benrose 2011, South Africa). Induction time averaged seven minutes. Elephants that fell in a sternal position were pulled into a lateral position. Their vital physiological parameters were monitored and once the elephant was declared stable the detusking process started. The full length of the tusk was measured from its tip to the point of skin contact and two thirds of the tusk was cut off using a power saw (Fig. 2). A third of the tusk was left to ensure that the central nerve was not exposed. Petroleum jelly was applied to the cut surfaces of the tusks to prevent cracking and chipping. Subsequently, the animals were revived with a Diprenorphine hydrochloride (M5050®) (Norvatis, South Africa) in the ear vein at three times the dose of the etorphine injected initially. Elephants were back up on their feet in an average of four minutes.

### Statistical analysis

A nonparametric ANOVA test (two-sample Mann Whitney Wilcoxon test) using an R Package V.2.15.1 [28] was applied to test the statistical significance of detusking on each elephant's fence-breaking rate before and after detusking. To test for possible seasonal differences in fence-breaking events before and after detusking the same test was applied separately for the data collected in the wet season and dry season. The wet season runs from March to May and October to December, while dry season includes January to February and June to September. Fisher's Exact Test, using the same statistical package, was applied to compare the seasonal differences in fence-breaking frequencies in the whole elephant population.

### Ethics

The Committee of the Department of Veterinary and Capture Services of the Kenya Wildlife Service (KWS) approved the study and all the animal capture and treatment protocols. KWS guidelines on Wildlife Veterinary Practice-2006 were followed. All the KWS veterinarians complied with the Veterinary Surgeons and Veterinary Para-Professionals Act, 2011, that regulates veterinary practice in Kenya.

## Results

During the study period (2010 to 2013), we registered 1041 fence-breaking incidents by elephants. We were unable to identify the culprit elephants in 21.61% (225/1041) of cases – “unknown fence-breakers” – but were able to identify culprits in the rest of the incidents (78.38%; 816/1041) – “known fence-breakers”. The fence-breakings by unknown fence-breakers were randomly distributed during the study period. Within the “known fence-breakers” we identified 67 (10 adult males and 57 adult females) elephants, 13.4% of a total of about 500 elephants in the Conservancy, that broke fences at least once during the study period. Assuming that the cases of fence-breaking carried out by unknown fence-breakers were performed randomly by male and female elephants (with the same percentages as for the known cases), the majority of fence-breakers were adult females, (57/67; 85.07%) and the others (14.93%; 10/67) were bulls. Female fence-breakers constitute 16.28% (57/350) of the total female elephants in the population, while male fence-breakers constitute 6.7% (10/150) of the total male elephants in the population. Nevertheless, these bulls were responsible for the majority of fence-breaking incidences (94.85%; 774/816) and females were only responsible for 5.15% (42/816) of the incidents. Fence-breaking turns out to be an exceptional ability acquired by specific bull elephants, which represent only about 1% (10/500) of the elephant population in the area (Table 1). Females carried out 76.2% (32/42) of their fence-breakings in the dry season and 23.8% (10/42) in the wet season (Fisher's Exact Test; *p* = 0.023); for males, 45.6% (343/774) of incidents were in the dry season and 54.4% (421/774) in the wet season (Fisher's Exact Test; *p* = 0.084).

**Table 1:** Number and sex of the known destructive elephants in Lewa Wildlife Conservancy; number and percentage of their fence-breaking activities during the study period 2010–2013; number and percentage of the incidents performed in dry and wet seasons.

Sex of fence-breaker elephants	Number of fence-breaker elephants (% from its total)	Number of fence-breakings (% from its total)	Number of fence-breakings in dry season (% from the total fence breaking of the same elephant sex)	Number of fence-breakings in wet season (% from the total fence breaking of the same elephant sex)
Females	57 (85.07)	42 (5.15)	32 (76.2)	10 (23.8)
Males	10 (14.93)	774 (94.85)	353 (45.6)	421(54.4)
Total	67	816	385	431

The real total number of fence-breakings was 1041, but 21.61% (225/1041) of incidents was performed by unknown fence-breakers. In our study we assume that the unknown cases were performed randomly (with the same known percentages) by male and female elephants.

Fence-breaking by the fence-breaker elephants gave access to other non-fence-breaker elephants, which can then presumably access the area once the fence is knocked down. When the fence-breaker is a matriarch, in most cases the entire family accompanies the matriarch; the numbers of the companions is 12±6, depending on family size. If the fence-breaker is a bull, the companion group is smaller (6±6).

Of the 10 bulls, eight named as Mountain Bull, Right Notch, Monk, Javet, Bullet, Keke, Flynn and Mshauri were identified as persistent fence-breakers. The technique used by these fence-breakers was always the same: they used their tusks to push wires up and down until they snapped. The average fence-breaking rate was up to four incidents per elephant per hundred days.

Most of the damage (75%; 780/1041) was to the two-stranded exclusion zones erected to exclude mega-herbivore animals (mainly elephants) and preserve browsing for black rhinoceroses. This type of fencing allows smaller game to pass underneath. The remaining damage was to the line of the main boundary fence.

The exclusion zones that were most prone to fence-breaking were Karionga and Willy Robert. The voltage on these fences was maintained above 5 kV. The main boundary fences damaged by elephants were sections near Kisima Farm, Ethi and Lodomoru villages. The Kisima Farm fence was either broken by elephants exiting or entering Ngare Ndare Forest to or from Mt Kenya Forest, or while invading wheat fields. Both the Ethi and Lodomoru fence lines, which protect agricultural smallholdings, were damaged by elephants attracted by wheat, carrot, maize and potato crops.

**Figure 3:**
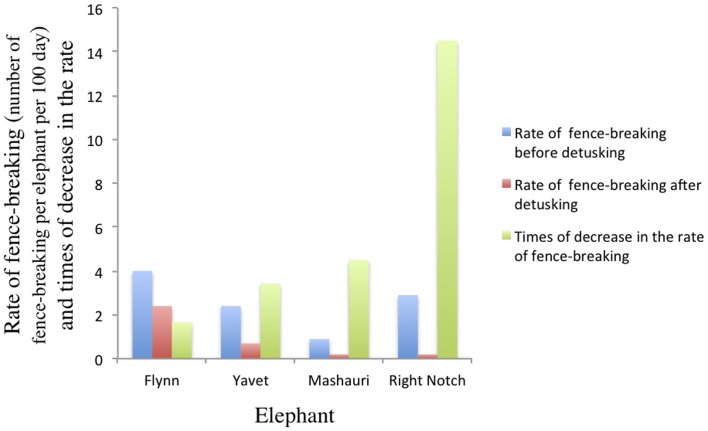
The relative reduction (times) in the fence-breaking rate of the detusked elephants before and after detusking. Elephants presumably used their trunks or legs to fence-break after detusking, or even the remaining parts of their tusks.

Four of the most destructive bulls (Flynn, Mshauri, Javet and Right Notch) were detusked during the study period once the KWS had granted permission. After detusking, the rate of fence-breaking (number of incidents per elephant per 100 days) was 1.67–14.5 times lower and the mean rate of attack fell six-fold (Fig. 3). The statistical analysis shows significant differences in the rate of fence-breaking before and after detusking in all detusked elephants (U-Mann Whitney test; W = 1090, *p* = 0.0151), affecting similarly both wet (U-Mann Whitney test; W = 1010, *p*<0.001) and dry (U-Mann Whitney test; W = 432, *p*<0.001) seasons in the same way.

## Discussion

The space left for nature conservation is becoming increasingly small and has led to a reduction in the range available for wildlife. This inevitably leads to increased HEC as elephants compete for grazing and watering points with humans and livestock [29]. Electric fences can reduce this chronic conflict by ensuring that human-elephant coexistence is possible and are considered as one of the most viable long-term options for controlling HEC [30].

Detusking elephants was tried a number of years ago in Kenya but was not accompanied by any proper study design or organised data collection [31]. Our data show that fence-breaking is not practiced by all the elephants that range near fences; rather, it is a habit acquired by just a few elephants that sometimes break fences. Only 13.4% of the total elephant population in the Conservancy damaged fences one or more times during the study period. Curiously, the majority of fence-breakers were adult females, although bulls are the cause of the majority of fence-breaking incidences; thus fence-breaking would seem to be an exceptional ability acquired by specific bull elephants, which represent only 1% of the total elephant population in the area. Our results concur with the findings of Chiyo et al. [4], who reported that fence-breaking and crop-raiding seem to be sex-biased towards males and probably depend on nutritional advantages that enhance their fitness and reproductive competitiveness [32].

Our results also show that fence-breaking is more prevalent among adult than young elephants. All the bulls and cows identified as fence-breakers were adults. This is probably due to the complex social relationships that exist in the tightly led matriarchal core units that offer security to young elephants, which contrast with the more flexible male units [2].

Not all alpha females or males are problematic, even when they share territories with humans and cultivation; likewise, not all members of an elephant group are prone to attack human settlements or cultivation [2]. Hence, a better understanding of this problem of elephant behaviour is of pivotal interest for future management plans and will help determine the correct response to such attacks.

Although female elephants broke more fences in the dry season than in the wet season, it seems that there is no seasonal pattern in the frequency of fence-breaking by bull elephants. This seasonal variation in female behaviour could be related to a real need for food to feed their calves; on the other hand, fence-breaking has become a habit (rather than a real need) for some elephant bulls and hence there is no seasonal variation in bull fence-breaking.

After the fence-breaker elephant has knocked down a fence, other non-fence-breaker elephants can access the area. It seems that preventing fence-breaking by one large male by detusking may prevent damage caused by several others.

One great advantage of detusking is that it reduces destructive activities aimed at the main fence lines and the exclusion zones (six-fold lower fence-breaking rate after detusking). After detusking, fence-breaker elephants lacked the tools they use to break fences. However, our study had certain limitations, one of which was the small sampling size (four bull elephants).

Moreover, other factors such as seasonal fluctuations were not taken into account in our study, e.g. in vegetation, which would have made the elephants that were responsible for the reduction in the fence-breaking rate less interested in the breaking down of fences to reach crops at certain times of year [33].

**Figure 4:**
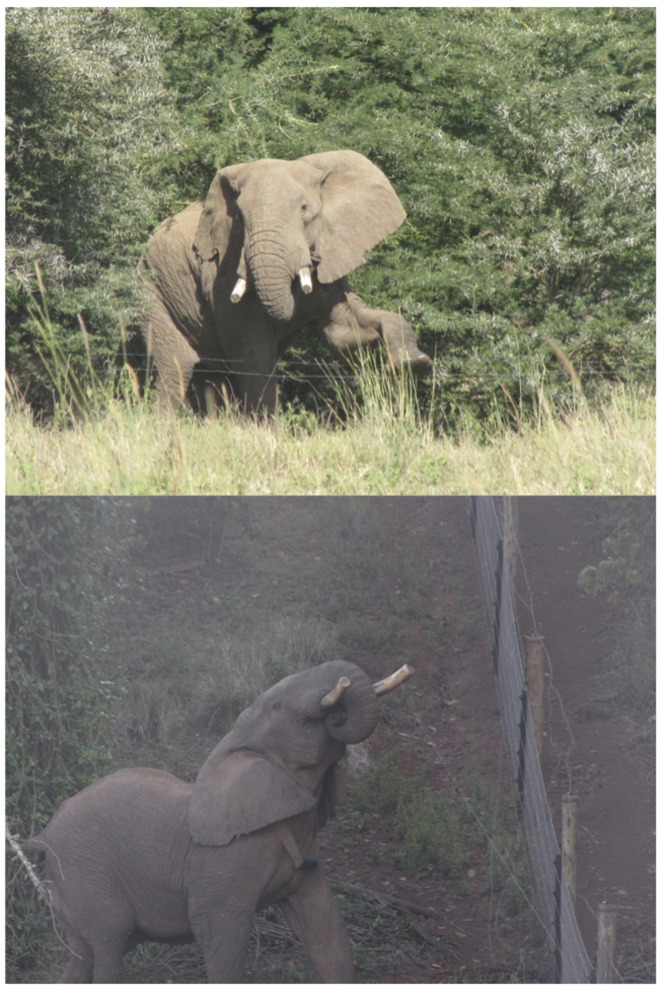
(Top) Right Notch (fence-breaker elephant) breaking an electric fence after detusking using his legs and trunk. (Bottom) Mountain Bull attempting to break an electric fence after detusking using the shortened tusks.

Detusking did not totally stop destructive elephants from damaging fences, probably due to the fact that, despite preferring to use their tusks to break down electric fences, fence-breakers can still perform their attacks using their heads, bodies and trunks. A number of opportunistic observations of detusked elephants show cases of post-detusking learning. Detusked elephants broke fences (i) using their fore-legs and trunks to flatten poles and then walk into the exclusion zone or, in some cases, (ii) using their shortened tusks to knock over the posts and the electric wires (Fig. 4). This latter behaviour is less frequent, since elephants receive electric shocks in the process. However, these sporadic observations do explain why detusked elephants did not totally stop breaking down fences. The most common ploy is to use the tips of the tusks, which do not contain any nerves, to snap wires.

Elephant tusks are a ‘trademark’ of this charismatic species and their size are a great indicator of an individual's genetic strength and viability [34]. Elephants are polygamous in nature and breeding and mate selection is based on physical dominance [35]. Elephant tusks are an excellent indicator of strength and a vital tool in the duels that determine the dominant bull in a group. They are used for digging up roots, stripping bark off trees for food, fighting during mating season and defending themselves against predators. The absence of tusks has the potential to degrade a virile male to second-rate status and to reduce his chances of breeding in a group. This could also affect the whole elephant population by allowing less virile males to pass on their genes instead of the once more dominant males [36], [37]. It has been shown that in some wildlife species such as bighorn rams (*Ovis canadensis*) the effects of chemical immobilization lead males – in spite of a full recovery – to lose their social rank [38]. Whether this is the case for elephants requires further investigation.

Minerals are a nutritional necessity in the diet and life of elephants [39]. Matriarchs occasionally lead entire herds to well-known salt licks or saltpans located along migratory routes or on riverbanks. These minerals play vital components in bone and tusk formation and complement diets [40]. The absence of such minerals from diets leads to a deficiency in minerals such as calcium and phosphorus and thus weakens bones. An elephant without tusks or with short or detusked tusks cannot dig out the essential minerals from the ground and is liable to suffer from mineral deficiency [26].

**Figure 5:**
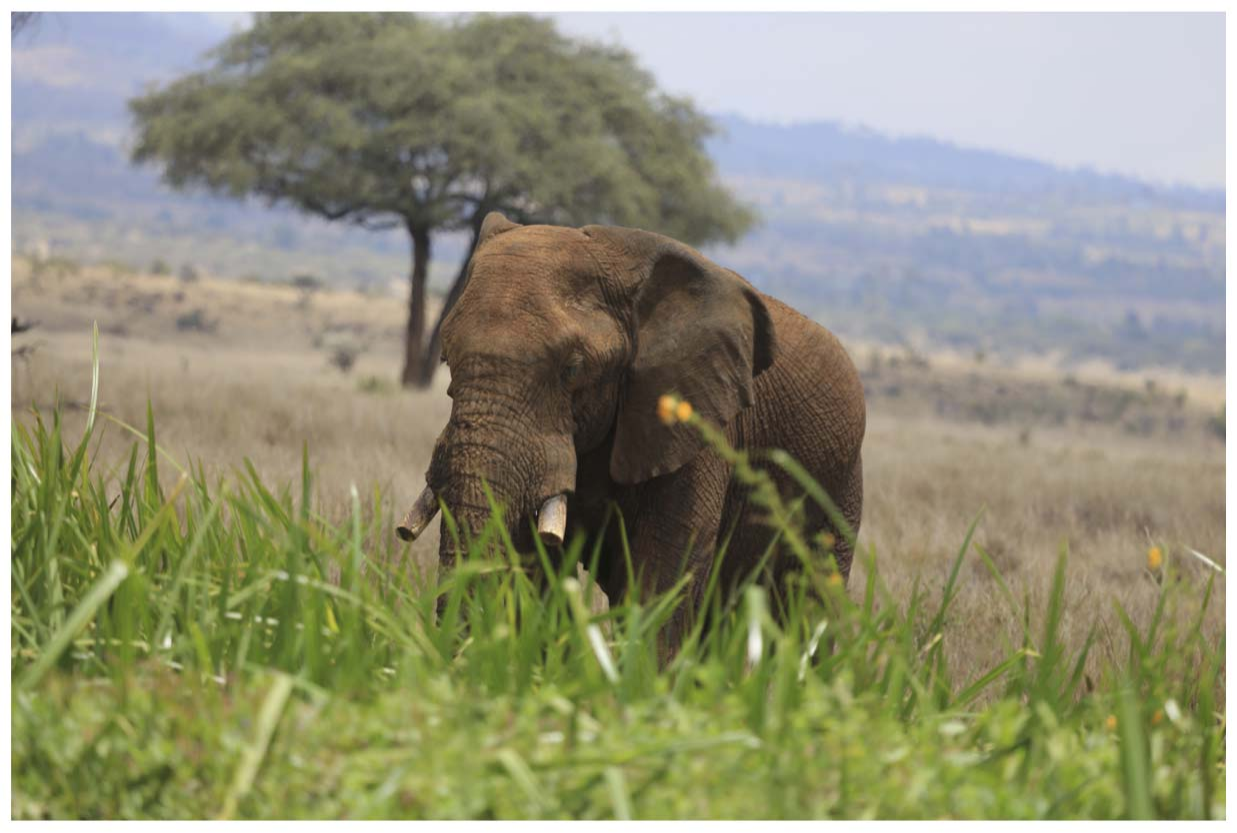
Photo of Mshauri (fence-breaker elephant) after detusking walking in Lewa Wildlife Conservancy.

Elephant tusks have a peculiar anatomical hook at their tip, an adaptation that is due to their prolonged when breaking branches and debarking trees. Tusks make elephants more effective feeders in woody mountainous and savannah environments in the dry season and better at defending their calves from predators [41]. The trimming of two thirds of the tusks thus deprives elephants of a vital feeding accessory. Moreover, the aesthetic value of an elephant is reduced greatly when it loses its natural body appendages [Fig. 5]. Detusked animals appear different and unauthentic to human-perceived attractiveness [27].

The detusking of fence-breaker elephants was successful in reducing fence-breakings and hence has helped mitigate human-elephant conflicts. Further studies are needed to test the efficacy of this method in terms of other aspects of this conflict such as crop-raiding by elephants, which is one of the most significant sources of human-elephant conflict.

## Conclusions

Elephant detusking is a relatively new approach being applied by the KWS on a very limited scale in extreme cases of fence-breaking. It aims to deprive some destructive elephants of their favourite tool for damaging fences. Elephant detusking is effective in drastically reducing the attacks performed by destructive elephants, but its negative effects on elephant behaviour, their ability to access food and the aesthetic consequences still need to be studied and discussed further.
